# Changing the organizational culture to transform the economy: The case of Greece

**DOI:** 10.3389/frma.2022.1050544

**Published:** 2022-12-07

**Authors:** Paraskevi Boufounou, Maria Despoina Argyrou

**Affiliations:** Department of Economics, National and Kapodistrian University of Athens, Zografou, Greece

**Keywords:** organizational culture, reforms, economic transformation, Hofstede analysis, OCAI indicators

## Abstract

**Introduction:**

Organizational culture determines the ability of companies to adapt, transform, and innovate, thereby directly affecting their profitability and competitiveness. However, the same applies to the public sector since, now more than ever, it has to be agile in order to shield its society and economy against modern challenges (such as COVID-19, climate change, and digitalization). This article uses the case of Greece as an example to present the need for change in organizational culture to unlock its development and growth potential through transformation, adaptation, and innovation. To support our argument, we combine the findings of the international literature regarding the relationship between organizational culture and the aforementioned elements, as well as empirical evidence from Greece.

**Methods:**

In particular, we assess organizational culture pertaining to the major sector reforms that took place in Greece, as a result of the recent economic and financial crisis, by presenting and evaluating comparative empirical findings on the characteristics of the prevailing and desired future organizational culture.

**Results and discussion:**

Examining and comparing the results of previous studies in Greece that used the Organizational Culture Assessment Instrument (OCAI) questionnaire survey in both private (such as banking and telecoms) and public sectors (such as social security, public revenues authority, and hospitals), two interesting results arise: (i) there is a clear distinction between the public sector and the private sector, with the former being mainly characterized by “hierarchy culture,” while the latter by “market culture” and (ii) in both sectors and all industries/services, the desire to prevail in future organizational culture is the “clan culture.” These findings are of immense importance as organizational culture issues play a key role in formulating future strategic plans, enabling the development of key sectors of the Greek economy and enhancing effective governance and social services. Concisely, our results draw useful conclusions for policy implications and academics, implying that there is an emergent need for organizational transformation in both private and public sectors in Greece, which can be achieved through new innovative methods of organization and operation, creating a new more agile, adaptive, and innovative culture.

## Introduction

The financial crisis of 2010 forced Greece to restructure both the public and private sectors, while COVID-19 enforced the adaptation of a new business model for many companies and organizations, accelerating the need for digitalization and adaptation of new working models and management techniques. However, the competitiveness of the Greek economy remains low compared with other countries. The present study aims to strengthen the argument that organizational culture is a key determinant that would enable the Greek economy to unlock its potential to reform, innovate, and grow.

Organizational culture determines the ability of companies to adapt, transform, and innovate, thereby directly affecting their profitability and competitiveness. The same applies to the public sector since, now more than ever, it has to be agile to shield its society and economy against modern challenges (such as COVID-19, climate change, and digitalization). In particular, the prevailing organizational culture affects the innovation capacity of entities through two channels: (a) by creating (or not) an enabling environment that promotes creativity, research, risk-taking, and experimentation, which also invests in new technologies and methods, and (b) by its effect on the overall performance of the organization, which affects its profitability and thus the available funds to invest for innovation. In other words, organizational culture affects the innovation level of organizations directly through its impact on the daily function of the organization and indirectly through its impact on the profitability of the firm, that is, on the available funds to invest for such purposes.

Hence, in the following section of the article, we summarize the results of previous studies that support our case with empirical evidence. There is a plethora of research studies showing a significant relationship between organizational culture and the overall performance and efficiency of the company, and employees' productivity and loyalty. Moreover, the organizational culture affects the adaptiveness of the company, that is, the way its people perceive change, their willingness to adopt new methods and technologies, and their ability to adjust to reforms and overcome challenges. Also, some organizational cultures promote innovation (e.g., clan culture), while others prioritize stability and control (e.g., hierarchy culture).

Especially for the case of Greek organizations, adaptiveness and innovation are vital elements, given that entities in Greece should evolve to survive in this modern digital era and the emerging new global sociopolitical and economic *status quo*, while at the same time, financial restrictions force them to be more and more innovative to achieve it. So, in the third section of this article, we describe the importance of culture for Greece at the current juncture of time, after a decade of economic recession.

In the next section, we present our methodology and perform a comparative analysis of the Organizational Culture Assessment Instrument (OCAI) surveys in five of the most important sectors—both private and public—of the Greek economy to identify patterns regarding the organizational culture. Subsequently, we discuss our results, attempt to rationalize our findings, and propose ways to implement the widely demanded change in the organizational culture. In the last section of the article, we summarize our results, present the limitations of our research, and suggest questions for future research.

## Literature Review

Since the 1980s, organizational culture has been considered one of the main variables affecting organizational performance (Pettigrew, 1979). Its importance increased over the years, and now, it is broadly considered one of the most important factors influencing organizational performance (Ahmed and Shafiq, 2014). Recent management research mainly focuses on aspects related to organizational culture issues and techniques, leading to significant results, such as the latest important concept of agile transformation.

Several researchers have tried to define organizational culture over the years (Titiev, 1959; Pettigrew, 1979; Kotter and Heskett, 1992; Stewart, 2010; Ahmed and Shafiq, 2014). However, for the purposes of this study, we used Schein's (1990) definition, according to which culture can be defined as “a pattern of basic assumptions invented, discovered, or developed by a given group as it learns to cope with its problems of external adaptation and internal integration that has worked well enough to be considered valid and therefore is to be taught to new members as the correct way to perceive, think, and feel in relation to those problems.”

Organizational culture is crucial for the operation and even the existence of organizations as Schein (2010) says, “If you do not manage culture, it manages you, and you may not even be aware of the extent to which this is happening.” In addition, much of the interest in the topic springs from the hypothesis that certain organizational cultures are associated with an improvement in organizational performance (Ilie and Gavrea, 2008). Organizational culture contributes to managing and coordinating organizational activities (Day, 1994), shaping procedures within the organization (Jarnagin and Slocum, 2007), providing solutions to many of the problems of the entity (Schein, 1984), and overall either hindering or facilitating its long-term goals (Denison, 1990).

Moreover, organizational development could increase effectiveness, while at the same time, improved performance leads to an increase in employee's commitment (Awadh and Saad, 2013). Thus, for many researchers, the organizational structure is the most important factor for organizational behavior, with a great direct impact on the overall performance of the organization (Kilmann et al., 1985; Ouchi and Wilkins, 1985; Schein, 1990).

In particular, in the private (for-profit) sector, organizational culture is essential for the success of a company, given its positive impact on the sustainability of the competitive advantage (Barney, 1986; Hall, 1993), through its effect on productivity (Umrani et al., 2017). A successful organizational culture promotes ethical behaviors, teamwork, and cooperation, while at the same time, it expands employees' efficiencies and self-confidence, leading to a consequent increase in productivity (Deal and Kennedy, 1982; Ouchi and Wilkins, 1985). Empirical findings suggest that organizational culture is among the main management factors (total quality management, business process re-engineering, knowledge management, leadership, etc.) affecting the overall performance of a company (Hansen and Wernerfelt, 1989; Sinclair, 1993; Wallace and Weese, 1995; Jung and Avolio, 1999; Detert et al., 2000; Lewis, 2002).

On the other hand, organizational culture is equally crucial for the public (non-for-profit) sector since an employee's happiness is crucial for its performance. If employees are positive about their working environment (which is directly affected by organizational culture) as it facilitates their most important needs at work, their morale increases, leading to better organizational performance. If this is not the case, that is, if employees are unhappy about their workplace, their morale decreases, resulting in lower productivity of the organization (Arunchand and Ramanathan, 2013). Public organizations operate in the same fast-pacing, complex, and volatile environment as private companies, but at the same time, they are, in general, more bureaucratic and less adaptable because of both their size and resources. As a result, organizational culture is vital for the public sector to be able to overcome the critical political, economic, and social challenges of today and tomorrow, such as globalization, digitalization, managerial growth, and geopolitical tensions (Lovell, 1995; Stewart and Kimber, 1996; Rukh and Qadeer, 2018). In order for the public sector to survive these turbulences and to continue serving society or even to improve the quality of its services, it has to enhance its efficiency, accountability, and transparency, which is only possible through changes in organizational culture (Boyne and Meier, 2009). Nevertheless, despite the importance of organizational culture in the public sector, the vast majority of studies examine its type and influence on the private sector; hence, there is a scarcity of empirical findings regarding the organizational culture of public organizations (Hood, 1991). A primary reason is that assessing organizational culture in public organizations is a very complicated and challenging task (Rukh and Qadeer, 2018).

In addition, cultural change is also more challenging in the public sector since no well-established organizational and professional culture exists in general, which, according to Brunetto (2001), is a facilitating factor of the transition. Based on empirical evidence, solutions regarding organizational practice proposed by the management and public choice theory have little to no success when implemented in the public sector, thus continuing to reflect traditional approaches to public administration (Parker and Bradley, 2000). In particular, findings from the French public sector support that “perpetual change is often perceived by the individuals as a generator of chaos and anomia, i.e., a loss of meaning for the subject who has lived in one organizational world, and who is asked to adopt the practices, norms, and behavior of another. This explains the resistance that human beings offer, which senior managers most often perceive as irrational and anti-progressist” (Fronda and Moriceau, 2008). Hence, it is proposed to use a “training and leading by example” approach, which can encourage cultural awareness and facilitate better cultural change in public organizations (Schraeder et al., 2005). This approach may be extremely useful for implementing the needed transition in countries with limited experience, like Greece.

Given the increasing significance of culture for the performance of both private and public sectors, more and more organizations attempt to change, shifting their organizational culture to a more efficient one. Successful leadership is a key factor in the process of cultural change, which “requires leaders to think culturally, to be guided by a cognitive model of change, and to employ the cultural tools of symbolism while actively focusing on the politics of acceptance” (Brooks, 1996). However, even in the case that successful leadership exists, this attempt is determined to fail if an organizational culture diagnosis (recognize the existing and identify the preferred one) has not been performed or if its results have not been analyzed (Ibid). Consequently, organizational culture diagnosis is an indispensable step for the organizational culture transition. Organizational culture diagnosis is a complex process that considers historical and contemporary elements as well as human perspectives. The final diagnosis is a subjective matter; hence, two different investigators may interpret the same results differently (Sathe, 1983). The diagnosis is made at multiple levels: culture as expressed by systems, processes, decisions, behaviors, shared values, and deeper principles concerning general beliefs about the world and perceived culture. Therefore, the first step is to define the conceptual framework, which may affect the overall image of the culture or specific elements of it, as well as the measurement tools, such as observational data, interviews, or questionnaires.

Earlier studies focused on elements of anthropological tradition, which formed an ideological background and thus shaped culture (Peters and Waterman, 1982). Such elements were the physical layout of the company space, company forms, publications in the press, company slogans, internal-use magazines and manuals, the existence or non-existence of company organizational charts, and finally various stories, myths, and events that have become part of the legacy of the corporate. Schein (1999) attempted to assess culture by interviewing selected groups to identify and understand intra-company views. Cameron and Quinn (1999) developed OCAI, which used a questionnaire to classify the organizational culture into one of the following types: clan, adhocracy, hierarchy, and market.

Recently, OCAI is the most widely used tool to perform organizational culture diagnosis. Table 1 summarizes results from the international literature regarding organizational culture in the sectors we examine in the Greek case.

**Table 1:** International empirical evidence of OCAI studies in the sectors of our analysis.

**Type**	**Sector**	**Study**	**Country**	**N**	**Present prevailing culture**	**Future preffered culture**
Private Sector	Telecoms	Gkila (2017)	Greece	150	Market	Clan
		Singh (2020)	India	375	Market	n/a
	Banks	Aldhuwaihi (2013)	Saudi Arabia	258	Market	n/a
		Belias and Koustelios (2014)	Greece	240	Hierarchy	Clan
		Jono (2016)	Indonesia	108	Clan	Clan
		Lampos (2016)	Greece	157	Market	Clan
		Naveed et al. (2016)	Pakistan	450	Hierarchy	Clan
Public sector	Public revenue authority	Tsegkou (2018)	Greece	179	Hierarchy	Clan
	Social security	Vassilakopoulou (2017)	Greece	152	Hierarchy	Clan
	Healthcare services	Van Huy et al. (2020)	Vietnam	566	Market/Clan	Clan
		Zervea et al. (2021)	Greece	160	Hierarchy	Clan
	Other budapest business school 3 ministries organizations of urban planning Marine department	Chandler et al. (2017)	Hungary	625	Hierarchy	Clan
		Nema (2021)	India	200	Hierarchy	Clan
		Rukh and Qadeer (2018)	Pakistan	444	Hierarchy	Clan
		Slack and Singh (2018)	Fiji	93	Hierarchy	Clan

As shown in Table 1, all researchers concluded the same result, that is, clan culture is the preferred one. Also, it is worth mentioning that in all studies reviewed, employees preferred a different organizational culture from the existing one, except in the case that the present organizational culture is already the clan culture (Jono, 2016).

## Organizational culture: The case of Greece

The present study assesses organizational culture pertaining to major sector reforms taking place in Greece, as a result of the current economic and financial crisis. More specifically, the empirical findings of OCAI questionnaire surveys carried out among employees in the private sector (such as banking and telecoms) and the public administration (such as social security, public revenues authority, and a public hospital) are presented and compared. The findings are important to effectively address impediments that enhance inertia and provide proper incentives that improve capacities and strengthen the alignment of the organization to the management change of the country, with an ultimate aim to improve national competitiveness in order to increase economic growth and, later on, human development.

To better understand the broader environment in which the sectors examined operate, we first try to sketch out a concise picture of the Greek economy. The overconsumption of the public and private sectors and the parallel loss of competitiveness of the Greek economy led to huge deficits in the current account balance, especially after 2000, which, in order to be serviced, led to an increase in external debt as a percentage of GDP from 40 in 2001 to around 85% in 2009 (Hellenic Statistical Authority, 2022). Combined with the simultaneous increase in interest payments on external debt, the country reached the brink of bankruptcy in 2010. Consequently, since 2011, the European Union has imposed several reforms to transform the Greek economy, improve national competitiveness, decrease public spending and public debt, and mitigate the risks of future crises.

In the end of 2019, the Greek economy rebounded, with economic and social life returning to normality. But this period of “happiness” did not last longer. COVID-19 hit Europe in January 2020, having a severe impact worldwide; Greece could not be an exception. This new crisis set new obstacles for organizations, forcing them to swiftly transform and adapt to the new reality and modern needs.

In this fast-changing, vague, and threatening environment of uncertainty and recession, organizations in Greece had to implement many changes in order to meet the new *status quo*, created by both formal (institutional reforms) and informal (change in customers' behavior) alterations. Adaptiveness and innovation are prerequisites for survival in this modern digital era and the arising sociopolitical and economic *status quo*. In this context, organizational culture is not a matter of consideration only for big entities since small Greek firms are often very dynamic and innovative, highlighting the need for a transition to a more agile culture and management. It is worth mentioning that SMEs are the main owners of the granted patents in Greece and hence the main producers of innovation in the country (Markatou, 2012). Moreover, in the previous years, the average size of Greek companies has increased. In 2012, 96–75%—of the Greek companies had up to nine employees, while the respective number in 2020 decreased to 94.94% (27,731 fewer companies). On the other hand, in the same period, the number of companies (in terms of employment) operating in Greece increased for all other company sizes, that is, an increase of (i) 7,657 companies with 10–19 employees, (ii) 3,856 companies with 20–49 employees, (iii) 750 companies with 50–249 employees, and (iv) 59 companies with over 250 employees (Eurostat, 2022).

Organizational culture was a key factor in this transition since depending on its type, it can either support or undermine the transformation process, as explained later. Hence, all sectors examined in our study have been through radical changes during the last 15 years. In short, the most important changes and reforms that have been implemented in these sectors since 2010 and the reasoning behind their selection are as follows:

**(A) Private sector**

- *Telecoms*: Since 2010, there have been no structural changes in the telecommunications sector. The mobile, internet, and telephony market are oligopolistic, consisting of three providers, whose market shares are practically stable in the last decade: Cosmote (~47%), Vodafone (~30%), and Wind (~23%). Only two notable deals took place in the sector in the last decade: in 2016, Vodafone acquired Hellas Online, and 3 years later, the same company acquired CYTA Hellas. We aimed to study the telecommunications sector because it is a key component for the growth of the Greek economy since it is part of the infrastructure required to digitalize the economy and transform the production model in order to meet the innovation 4.0 standards.
- *Banks*: The Greek banking system has experienced many alterations in the last 20 years. Following the course of the global banking system, a number of banking institutions suffered a gradual reduction, and mergers and acquisitions dominated. During the economic crisis, it became impossible for small banks to survive; therefore, they were acquired by larger ones. Also, a significant share of the banking market consisting of the so-called “Cypriot banks”, which, after the crisis in the banking system of Cyprus in March 2013, ceased their operations in Greece and—with government intervention—their branches in Greece passed into the hands of their Greek peers. Currently, four large systemic banks remain, whose cultural diagnosis is presented later. Moreover, the stability of the sector has drastically improved in the last years through the significant reduction of NPLs (from 25.5 in 2020 to 7% in 2021). Also, at the end of 2021, Greece became one of the first countries to adopt the new Insolvency Code, implementing the European Directive on Preventive Restructuring and Insolvency (IMF, 2022). The financial sector, especially banks, is one of the main pillars of the economy, the quality of which affects the whole economy. For this reason, we included the banking sector in our analysis.

(B) Public sector

- *Revenue Authority*: In 2017, the new public revenue administration organization named Independent Authority for Public Revenue was established by law 4389/2016. The new organization is much more independent than the previous organization (General Secretary of Public Revenue), following the international standards of tax administration autonomy and enjoying financial, operational, and administrative independence. In addition, the authority is subject to parliamentary scrutiny, but not subject to scrutiny or supervision by government bodies or other administrative authorities (IAPR, 2022). Public finance is a critical component of the national economy and public revenue is a big part of it. Thus, we could not exclude the Independent Public Revenue Authority from our study, since its performance has a great impact on economic policy decisions, and thus a strong direct effect on the whole economy.
- *Social Security*: In 2016, the Greek pension system experienced the biggest restructuring in its history. The key reforms included “the integration of all insurance funds into one agency, the replacement of the main pension by a national pension and a contributory pension, and the introduction of uniform rules and equal pension rights as well as measures for the containment of pension expenditure.” (Ziomas and Theodoroulakis, 2016). Hence, we examined the social security sector because it experienced one of the most radical reforms, while COVID-19 emphasized its value for society and the economy.
- *Healthcare Services*: The primary goals of the reforms in the healthcare sector were to improve the structural aspects of the system while, at the same time, enhancing spending efficiency. The main channels used to achieve these goals were the rationalization of pharmaceutical spending and public spending for healthcare, pricing policies, reorganization of primary and secondary healthcare networks, a merger of health insurance funds, harmonization of healthcare benefits, and changes in the system of copayments (Kalavrezou and Jin, 2021).
- The reasoning behind including the public healthcare services sector in our study is similar to the social security sector, that is, fundamental reforms have been implemented in this sector, while COVID-19 highlighted its importance for both society and the economy.

As shown in Table 2, these sectors have been selected because of their vital importance for the whole economy since they significantly contribute to both production and employment. Moreover, the selected sectors are the most affected by the reforms implemented in the last decade. Therefore, organizational culture is of high importance since it affects the ability of organizations to adapt, innovate, and eventually survive.

**Table 2:** Sectors' contribution to employment and production, 2019.

**Type**	**Sector**	**Companies**		**Turnover**		**Employees**	
		**Number**	**% of total**	**EUR thousand**	**% of total**	**Number**	**% of total**
Private sector	Telecoms	1.449	0,10%	5.907.916	1,90%	23.146	0,50%
	(Nace code 61)						
	Banks	106	0,01%	11.615.976	3,70%	40.145	0,90%
	(Nace code 641)						
Public sector	Public revenue authority	13	0,00%	133.139	0,00%	10.554	0,20%
	(Nace code 843)						
	Social security	1.03	0,07%	299.967	0,10%	192.632	4,30%
	(Nace code 841)						
	Healthcare services	856	0,06%	1.356.303	0,40%	113.348	2,60%
	(Nace code 861)						

Hellenic Statistical Authority, 2022.

Nevertheless, research on interpreting the behavior of Greek organizations based on organizational culture was rather sketchy, but current research using Hofstede dimensions is increasing (Veiga and Yanouzas, 1991; Theotokas and Progoulaki, 2007; Tsakumis, 2007; Tsoukatos and Rand, 2007; Kritikou et al., 2021). Thus, to outline the big picture, we present cultural characteristics at the country level based on the Hofstede Insights network analysis. Their findings support that cultural differences among nations are identified based on values, while differences among organizations are found based on practices (Hofstede, 1980; Hofstede et al., 1990).

**Figure 1:**
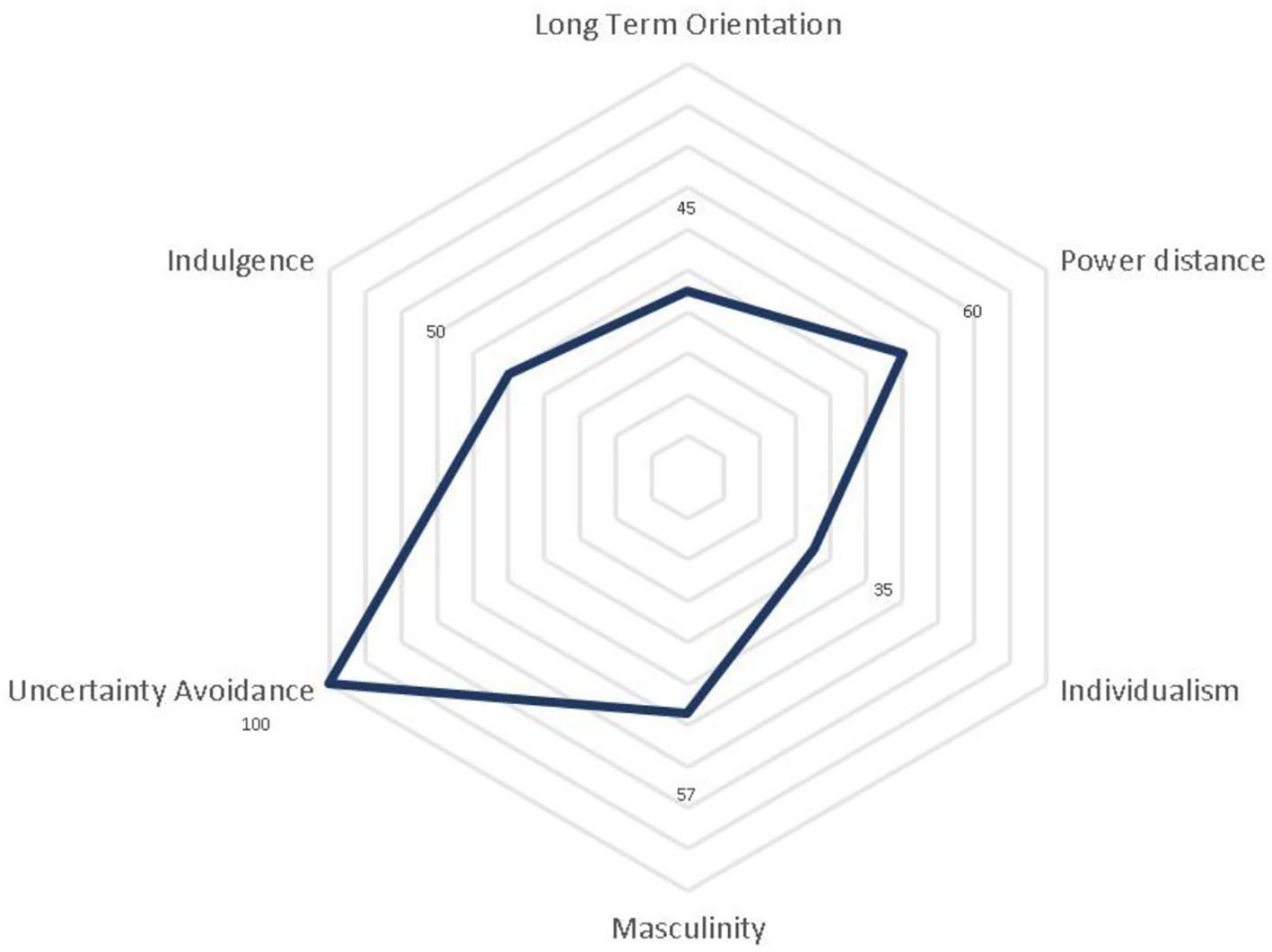
Cultural characteristics of Greece. Hofstede Insights. (2022) Country Comparison-Greece. Available at https://www.hofstede-insights.com/country-comparison/greece/ (accessed September 18, 2022).

In particular, as presented in Figure 1, regarding the cultural characteristics of Greece, according to the network analysis, the Greek culture is characterized by very high uncertainty avoidance, accompanied by low long-term orientation and individualism. Comparing the cultural characteristics of Greece with those of other EU countries based on the network analysis data, it is evident that uncertainty avoidance, power distance, and masculinity are stronger in the Greek culture, while long-term orientation, individualism, and indulgence are of higher importance for the EU average than for Greece.

## Materials and methods

Organizational Culture Assessment Instrument surveys classify the organizational culture into one of the following types: clan, adhocracy, hierarchy, and market. Clan culture emphasizes internal coherence, flexibility, and interest in people. Adhocracy culture emphasizes visibility and market positioning and promotes individuality, innovation, and flexibility. Hierarchy culture promotes stability and control through internal coherence. The market culture emphasizes visibility, market positioning, competitiveness, and productivity (Quinn et al., 1996). *Becoming a Master Manager: A Competency Framework*. Wiley.

- *Clan culture:* This culture shows an emphasis on human relations and the prevalence of a friendly atmosphere with elements of participation, teamwork, freedom of expression, loyalty, mutual trust, and harmony. The company embracing cult culture is interested in the development and training of employees in a friendly atmosphere.
- *Adhocracy culture:* This culture supports the ability and development of staff through initiatives, experimentation, innovation, and adaptation to changes in the external environment. The goal is to be ahead of the competition.
- *Hierarchy culture*: This culture is characterized by the observance of regulations, procedures, and hierarchy in order to control the individuals and the group and the stability of the structures.
- *Market Culture:* This culture emphasizes achieving results through task definition, rationalization, planning, performance standards, and efficient use of resources. Productivity, efficiency, and competitiveness are monitored through targets set by leadership.

Each type of culture is approached through a questionnaire that contains the six dimensions of culture, which according to Cameron and Quinn (1999), are the dominant organizational characteristics, leadership style, management of employees, organizational glue, strategic emphasis, and criteria of success.

The goal of OCAI is to capture the current organizational culture in each company that is used and also to capture the culture desired by the staff of each organization that will prevail in 5 years from the present. For this reason, six questions are used, each of which corresponds to a basic dimension of culture.

For each question, there are four alternative answers (one for each culture type). The respondent is asked to give a score from 0 to 100 to each answer according to how well the proposition of the answer fits their work environment, where 0 indicates no match at all and 100 indicates a perfect match. However, the sum of the points of all four alternative answers must be equal to 100. The same procedure is followed for the same questions concerning the future. Then, the average of each answer corresponding to the same culture is calculated (sum by 6), and the average of all the answers is obtained. In this way, a score is obtained for each type of culture (the sum of the four scores must be equal to 100). The culture with the highest score is the one that prevails or that the respondents would like to prevail after 5 years.

Organizational Culture Assessment Instrument is valid and reliable and is used successfully in many studies (Lamond, 2003). Thus, the following analysis uses the results of studies based on OCAI questionnaires as a diagnostic tool for assessing organizational culture.

As mentioned before, the purpose of the present study is to strengthen the argument that organizational culture is a key determinant that would allow the Greek economy to unlock its potential to reform, innovate, and grow. Thus, we performed a comparative analysis of the organizational culture of five sectors (both private and public) of the Greek economy, selected on the following basis:

a) Importance of the sector to the economy
b) Radical reforms have been implemented (or are expected)
c) Availability of reliable and trustworthy OCAI surveys.

Therefore, we based our analysis on the findings of the corresponding studies on five studies that we considered trustworthy^1^ and comparable as they are cross-sectional and comprehensive studies, which are described as follows:

a) ***Private sector:*** Two studies examined the culture by surveying 752 questionnaires in total, including 150 questionnaires on telecoms (Gkila, 2017) and 602 questionnaires on banks (Lambos,2016).
b) ***Public sector***: Three studies assess the culture by surveying 491 questionnaires in total, including 179 questionnaires on revenue authority (Tsegkou, 2018), 152 questionnaires on social security (Vassilakopoulou, 2017), and 160 questionnaires on healthcare services (Zervea et al., 2021).

Thus, we consider the samples as representative of the corresponding sectors; hence, the findings of the organizations examined in the aforementioned studies can be used as a proxy for the organizational culture of the whole sector they belong to. Table 3 presents the descriptive statistics of the OCAI surveys analyzed.

**Table 3:** Descriptive statistics of the surveys analyzed.

**Sector**		**Culture Type**	**Time**	**No**	**Mean**	**Std. Deviation**	**Min**	**Max**
Private sector	Telecoms	Clan culture	Present	150	10,90	0,82	10,00	12,84
			Future	150	**43,03**	1,25	40,50	45,00
		Adhocracy culture	Present	150	11,19	0,99	9,67	14,33
			Future	150	13,45	0,51	12,17	14,67
		Market culture	Present	150	**46,98**	1,44	45,00	49,50
			Future	150	14,53	1,69	11,33	18,00
		Hierarchy culture	Present	150	30,93	1,47	28,00	33,83
			Future	150	28,99	0,99	26,17	30,67
	Banks	Clan culture	Present	157	17,79	10,14	73,33	0,00
			Future	157	**30,03**	16,26	83,33	0,00
		Adhocracy culture	Present	157	18,04	6,85	34,33	0,00
			Future	157	24,33	8,95	60,00	0,00
		Market culture	Present	157	**37,04**	14,94	81,67	6,67
			Future	157	23,68	15,09	93,33	0,00
		Hierarchy culture	Present	157	27,03	10,36	80,00	3,33
			Future	157	22,38	8,18	57,50	3,33
Public sector	Public revenue authority	Clan culture	Present	169	19,37	10,13	0,00	59,17
			Future	179	**32,18**	11,46	0,00	75,00
		Adhocracy culture	Present	169	15,69	7,02	0,00	36,67
			Future	179	26,10	7,19	0,00	46,67
		Market culture	Present	169	27,52	10,30	8,33	65,00
			Future	179	22,36	10,43	3,33	66,67
		Hierarchy culture	Present	169	**37,43**	15,23	5,00	90,00
			Future	179	19,37	8,13	0,00	45,00
	Social security	Clan culture	Present	152	20,22	15,60	0,00	81,60
			Future	152	**35,41**	21,93	0,00	98,33
		Adhocracy culture	Present	152	12,10	11,39	0,00	70,00
			Future	152	26,77	15,65	0,00	90,83
		Market culture	Present	152	17,59	17,58	0,00	85,00
			Future	152	17,86	13,74	0,00	69,16
		Hierarchy culture	Present	152	**50,07**	25,40	0,00	100,00
			Future	152	19,95	19,94	0,00	81,66
	Healthcare services	Clan culture	Present	160	25,29	9,77	n/a	n/a
			Future	160	**32,67**	10,09	n/a	n/a
		Adhocracy culture	Present	160	18,69	6,33	n/a	n/a
			Future	160	22,97	7,42	n/a	n/a
		Market culture	Present	160	23,52	9,29	n/a	n/a
			Future	160	18,73	6,58	n/a	n/a
		Hierarchy culture	Present	160	**32,49**	11,82	n/a	n/a
			Future	160	25,63	9,60	n/a	n/a

Lampos, 2016; Gkila, 2017; Vassilakopoulou, 2017; Tsegkou, 2018; Zervea et al., 2021, processed by authors. The bold letters indicate the dominant culture type in every industry.

## Results

This section summarizes the main findings of the aforementioned surveys by sector, it is evidenced as follows:

***(A) Private sector***

- *Telecoms*: The market culture is the dominant type (~47%) of organizational culture in the Cosmote group of companies, which, for our research, represents the whole telecommunications sector. However, employees have a strong preference for the company to shift to the clan culture in the future (~43%). These findings are in line with those of Papadimitriou and Kargas (2012), who showed that the dominant cultures in the telecommunication companies in Greece are the market culture and the adhocracy culture since they better serve the demands of their competitive environment.
- *Banks*: The prevailing organizational culture in the Greek banking sector is the market culture (32.7%) accompanied by a strong presence of the hierarchy culture (30.6%). The employees would prefer to adopt more clan culture (28.6%) characteristics in the future, while the current dominant culture is the least desired one (22,7%). These results agree with those of the study by Belias and Koustelios (2014), who performed a similar study about a decade ago, indicating persistence in the difference between the existing and desired organizational culture in the Greek banking sector.

***(B) Public sector***

- *Revenue Authority*: The prevailing type of organizational culture in the Independent Public Revenue Authority is the hierarchy culture (~37,4%), but most employees would prefer a different organizational culture. In particular, the most favorable one is the clan culture (~32.2%), followed by adhocracy culture (~26.1%), while the least preferable is the current culture (hierarchy, ~19.4%).
- *Social Security*: The social security sector has a clear hierarchy culture (~50,1%), although the employees desire the clan culture to prevail in the future (~35,4%), followed by the adhocracy culture (~26,7%).
- *Healthcare Services*: The hierarchy culture (~32,5%) is the dominant type of organizational culture in public hospitals as well. However, in that case too, the majority of employees would prefer the clan culture (~32,7%) in the future, while the current culture (hierarchy) is the second most favorable (~25,6%). Our results are in line with those of the study by Bourntenas et al. (2014), who performed a similar study in the General Hospital of Larissa. However, comparing our findings with those of Bista et al. (2018) showed that there are differences in the current culture (in the latter, the dominant culture is the adhocracy culture but the desired culture is the clan culture, in this case as well). However, this contradiction does not undermine the reliability of our analysis since the two OCAI surveys took place in different organizations [Chania General Hospital in the study analyzed and Preveza Hospital in the study of Bista et al. (2018)].

**Figure 2:**
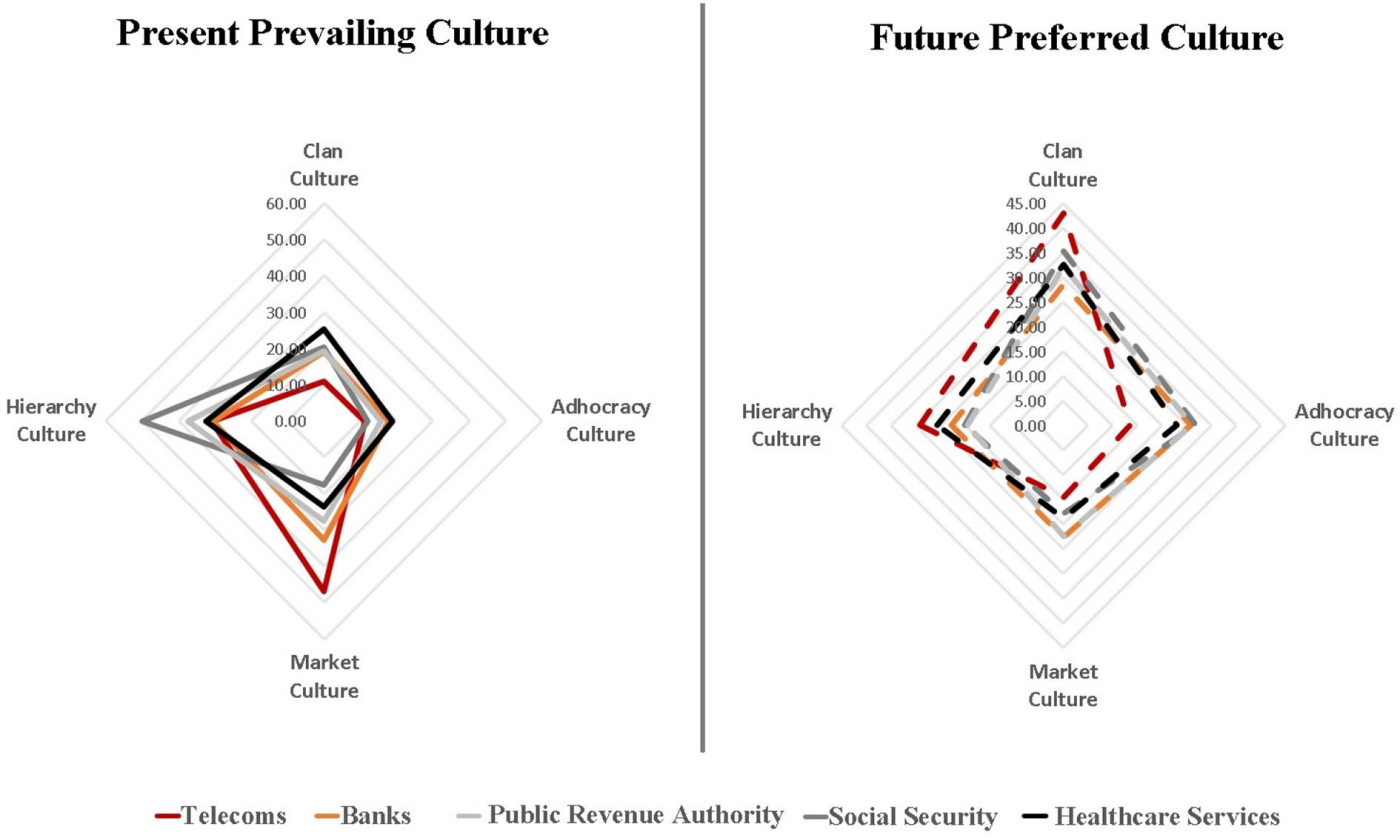
Current and desired organizational culture in five sectors of the Greek economy. Lampos, 2016; Gkila, 2017; Vassilakopoulou, 2017; Tsegkou, 2018; Zervea et al., 2021, processed by authors.

The final findings of the OCAI surveys analyzed, offering an overview of organizational diagnosis for five vital sectors of the Greek economy, are presented in Figure 2.

**Figure 3:**
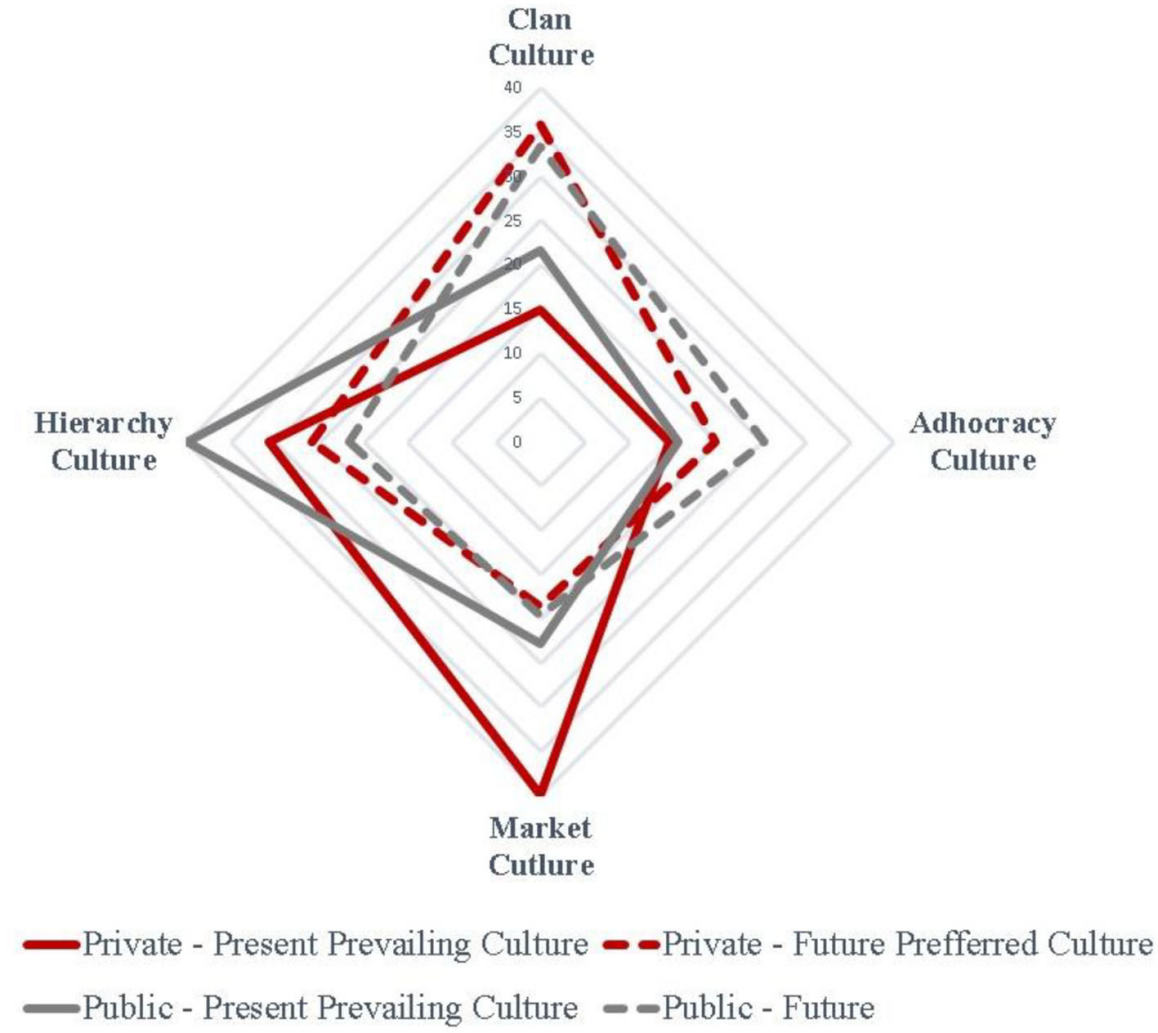
Current and desired organizational culture in private and public sectors of the Greek economy. Lampos, 2016; Gkila, 2017; Vassilakopoulou, 2017; Tsegkou, 2018; Zervea et al., 2021, processed by authors.

The market culture prevails in the Greek private sector, followed by the hierarchy culture, while in the public sector, the hierarchy culture is clearly the dominant culture. However, it is thought-provoking that all organizations examined, in both sectors, desire the clan culture to prevail in the next 5 years, as illustrated in Figure 3.

Our results comply with the findings of the international literature. First of all, the main conclusion is that the desired future culture in all sectors examined is the clan culture is common in both cases. Also, the empirical results of the literature review presented in Table 1 suggest that the present prevailing culture in the public sector is the hierarchy culture, with the exception of the study of Van Huy et al. (2020), while the preferred future culture is the clan culture, which is in line with our findings. However, there are differences regarding the present prevailing culture in banks since the international literature does not provide a clear answer regarding the present most common prevailing culture in the banking sector as the results are mixed.

The fact that the employees of all sectors would prefer a shift to the clan culture despite the type of the current dominant culture is very interesting since it questions the “*status quo* bias” (Samuelson and Zeckhauser, 1988), that is, the strong tendency individuals have to remain in the default option (Kahneman et al., 1991). However, this could be explained if we consider that the clan culture is people-focused, promoting collaboration and communication, which better matches the national cultural characteristics of Greece, as presented in Figure 1, while it is also more compatible with the contemporary needs of the society, which has redefined its priorities and radically transformed its lifestyle after the financial crisis of 2007.

## Discussion

The results of our analysis indicate that regardless of the sector, the ownership (public or private), and the type of the present prevailing culture, there is—from the employees' side—a strong desire for change in the organizational culture. This yearning for change contradicts the norm that people do not like and/or are afraid of changes; hence, they prefer to go with the default option, in other words, to maintain the *status quo* (Samuelson and Zeckhauser, 1988; Kahneman et al., 1991).

We suggest the reasons behind this strong demand for change in the organizational culture, which are as follows:

(d) *National cultural characteristics*: The cultural characteristics of Greek society (such as low individualism and long-term orientation) are more compatible with the clan culture, which is characterized by a friendly atmosphere, teamwork, freedom of expression, loyalty, mutual trust, and harmony.
(e) *Redefined life priorities:* The financial crisis of 2007 and the most recent healthcare crisis had a severe impact on society internationally. Not only consumers changed their behavior and preferences but also people redefined their priorities, needs, and definition of success and happiness. More and more employees prefer work–life balance over a higher salary. Success and happiness are now less related to monetary values since other factors such as free time, mental health, and human relationships are of increased importance. Given that employees spend about one-third of their life in their workplace, this new mindset emphasized the need for the creation of more human-centered organizations, which will embrace and reflect this new approach to life.
(f) *Crave for economic prosperity:* The Greek society and economy have undergone more than 11 subsequent years of crisis. The economic crisis of 2010 decreased the national GDP by more than 25%, while the economy slightly recovered between 2017 and 2019 (1.09–1.8% annual GDP growth rate, Hellenic Statistical Authority, 2022). However, the economy did not manage to take off since the global pandemic outburst in 2020, resulting in a 9% reduction in the national GDP. Greek people have suffered much. Many structural reforms and changes (institutional and non-institutional) have been implemented in the last 10 years, resulting mainly in the radical transformation of the public sector. However, the competitiveness of the Greek economy remains comparably low for a developed EU country. Hence, many people nowadays recognize that there are also factors inside organizations that reduce efficiency and productivity. So, employees now realize that the way organizations operate should change to unlock employees' potential and improve business performance which will lead to economic growth. In other words, people are both mentally and financially exhausted because of more than a decade of economic stagnation. Therefore, they acknowledge the need for change, and thus, they support, promote, and desire the adaptation of a new *status quo*. As explained earlier, the organizational culture is not only a factor that can increase employees' productivity and performance but also a more agile organizational culture that can facilitate the transformation of the company (adoption of new technologies, management models, etc.,). As a result, employees ask for a change in the organizational culture as they perceive it as a milestone for the overall evolution of the organization (Gallup, 2018).

Combining previous findings can explain the need to change the existing organizational culture to a new culture, which will better serve both the day-to-day needs of the employees and the long-term goal of economic growth by creating a more efficient and competitive production model. In addition, points (a) and (b) better rationalize the direction of this change, thus, the clan culture is the most favorable among Greeks. Also, point (b) could explain why the clan culture is so popular worldwide.

The previous discussion justifies the need of most of the Greek sectors to shift to clan culture. Hence, we would also like to propose some actions that can be easily implemented and can be taken to support the adoption of the clan culture in an organization:

- Establishment of the 360-degree evaluation system for all leaders, that is, their evaluation by their assistants, partners, and superiors.
- The design of professional development programs that enhance internal mobility and interdepartmental communication.
- Establishing research programs to help identify staff attitudes and ideas. Findings should be implemented by planned working groups.
- Increased involvement of staff in all stages of strategic planning.
- Development of programs to facilitate and increase the team spirit of the staff.
- Identification of long-term conflicts and disagreements within the departments and the design of interventions to overcome them.
- Development of procedures regarding the handling of employee diversity.
- Examining the expectations of middle managers and providing incentives to strengthen their powers.
- Assigning decisions about pay and budget management to lower hierarchical levels.
- Development of training programs for middle managers with the aim of better understanding the strategic processes and their role in terms of the effectiveness of the organization.

To sum up, we believe that the organizational culture is a crucial factor to achieve the long-awaited takeoff of the Greek economy, which could also facilitate and enhance the growth potential of the country. Based on the results of our analysis, there is a strong desire from Greek employees that their organizations shift to the clan culture. We argue that by applying modern methods, such as agile transformation, Greek organizations can smoothly transform their culture to the preferred culture, which, based on our findings, is the clan culture. If this transition is adopted on a significant scale, it could lead to increased productivity, efficiency, and innovation at the national level and hence to the overall growth of the Greek economy.

## Conclusion

In this article, we present, evaluate, and compare empirical findings on the characteristics of the prevailing and desired future organizational culture in five important segments of the Greek economy (private sector: telecommunications and banks; public sector: social security, Independent Public Revenue Authority, and public healthcare services). Our results show that there is a clear difference in the existing prevailing organizational culture between the private and public sectors. In particular, according to our sample, the organizational culture in the private sector is mainly based on the principles of the market culture, while in the public sector, the hierarchy culture is the prevailing culture. However, the most interesting result is that regardless of the sector (public or private), the industry, and the type of the present prevailing culture, the desired future organizational culture is the clan culture in all sectors examined. Also, we attempted to rationalize this unusual demand for change, which questions the empirically proven *status quo* bias and the default effect bias, by proposing that it springs from the cultural characteristics of Greece, the redefined life priorities and the desire for economic prosperity in the Greek society after a decade of recession.

However, our study has significant limitations mainly because of the small number of the organizations and sectors examined since, as mentioned earlier, reliable and thorough OCAI surveys in Greece are still in scarcity. Nevertheless, we consider that through our—precise, but not extended—comparative analysis, we produced results that show a clear pattern.

We expect that our research will intrigue other researchers to examine the differences between the organizational culture in the public and private sectors, the significance of the organizational culture in Greece, and the reasons behind employees' demand for organizational change. Future research should enlarge the number of organizations and sectors studied in order to validate the existence of the patterns evident in this article. Also, it would be very interesting to perform a similar analysis (including the OCAI surveys) 5 years from now, to examine the future impact on the sectors examined in this study, that is, if they will have shifted their culture closer to the clan culture (or not) and what will be the results of this transition (or inertia), especially regarding the success of the reforms implemented.

Moreover, given that our analysis examines data only for Greece—a very peculiar case—and the sociocultural environment has a direct strong impact on the internal work culture (Aycan et al., 1999), its results should not be generalized to other countries but rather used carefully as an indication. Thus, it would also be of great interest to perform similar studies in other countries, particularly in countries that implemented structural changes.

## Data availability statement

The original contributions presented in the study are included in the article/supplementary material, further inquiries can be directed to the corresponding author.

## Author contributions

All authors listed have made a substantial, direct, and intellectual contribution to the work and approved it for publication.

## Footnotes

1 The relative theses of the first four of them were supervised by the first author of the present paper, while the fifth one (on healthcare services) is published in a well-known journal by respectful researchers.

## References

[B1] Ahmed M. Shafiq S. (2014). The impact of organizational culture on organizational performance: a case study on telecom sector. Global J. Manage. Bus. Res. 14, 21–30. Available online at: https://journalofbusiness.org/index.php/GJMBR/article/view/1254 (accessed September 07, 2022).

[B2] Aldhuwaihi A. (2013). The Influence of Organisational Culture on Job Satisfaction, Organisational Commitment and Turnover Intention: A Study on the Banking Sector in the Kingdom of Saudi Arabia (Doctoral Dissertation) Footscray: Victoria University.

[B3] Arunchand C. H. Ramanathan H. N. (2013). Organizational culture and employee morale: a public sector enterprise experience. J. Strategic Hum. Resource Manage. 2. Available online at: https://www.academia.edu/32678444/ORGANIZATIONAL_CULTURE_AND_EMPLOYEE_MORALE_A_PUBLIC_SECTOR_ENTERPRISE_EXPERIENCE (accessed August 29, 2022).

[B4] Awadh A. M. Saad A. M. (2013). Impact of organizational culture on employee performance. Int. Rev. Manage. Bus. Res. 2, 168–175. Available online at: https://irmbrjournal.com/papers/1364462611.pdf (accessed September 02, 2022).

[B5] Aycan Z. Kanungo R. N. Sinha J. B. (1999). Organizational culture and human resource management practices: the model of culture fit. J. Cross-Cult. Psychol. 30, 501–526. 10.1177/0022022199030004006

[B6] Barney J. B. (1986). Organizational culture: can it be a source of sustained competitive advantage?. Acad. Manage. Rev. 11, 656–665. 10.2307/25831724396949

[B7] Belias D. Koustelios A. (2014). The influence of demographic characteristics of Greek bank employees on their perceptions of organizational culture. Int. J. Hum. Resource Stud. 4, 81–100. 10.5296/ijhrs.v4i1.5058

[B8] Bista Á. Prezerakos P. Moisoglou I. Dreliozi A. Platis C. (2018). Organizational culture and change: the case of a Greek public hospital. Int. J. Health Res. Innovat. 6. Available online at: http://www.scienpress.com/Upload/IJHRI/Vol%206_1_1.pdf (accessed September 03, 2022).

[B9] Bourntenas D. Kastanioti C. Niakas D. Tsouri M. (2014). The influence of organizational culture on job satisfaction of administrative employees at a public hospital: the case of general hospital of Larissa. J. Health Manage. 16, 217–231. 10.1177/0972063414526108

[B10] Boyne G. Meier K. (2009). Environmental turbulence, organizational stability and public service performance. Administrat. Soc. 40, 799–824. 10.1177/0095399708326333

[B11] Brooks I. (1996). Leadership of a cultural change process. Leadership Organiz. Develop. J. 17, 31–37. 10.1108/01437739610127496

[B12] Brunetto Y. (2001). Mediating change for public-sector professionals. Int. J. Public Sector Manage. 26, 492–502. 10.1108/09513550110408639

[B13] Cameron S. K. Quinn E. R. (1999). Diagnosing and Changing Organizational Culture. Boston, Massachusetts: Addison-Wesley.

[B14] Chandler N. Heidrich B. Kasa R. (2017). Everything changes? A repeated cross-sectional study of organisational culture in the public sector. Evidence Based H. R. M. 5, 283–96. 10.1108/EBHRM-03-2017-0018

[B15] Day G. S. (1994). The capabilities of market-driven organizations. J. Market. 58, 37–52. 10.1177/002224299405800404

[B16] Deal T. E. Kennedy A. A. (1982). Corporate Cultures Reading: The Rites and Rituals of Corporate Life. Boston, Massachusetts: Addison-Wesley.

[B17] Denison D. R. (1990). Corporate Culture and Organizational Effectiveness. Hoboken: John Wiley and Sons.

[B18] Detert J. R. Schroeder R. G. Mauriel J. J. (2000). A framework for linking culture and improvement initiatives in organizations. Acad. Manage. Rev. 25, 850–863. 10.2307/259210

[B19] Eurostat (2022). Number of Enterprises in The Non-Financial Business Economy by Size Class of Employment. Online data code: TIN00145.

[B20] Fronda Y. Moriceau J. (2008). I am not your hero: change management and culture shocks in a public sector corporation. J. Organizational Change Manage. 21, 589–609. 10.1108/09534810810903234

[B21] Gallup (2018). Gallup's Approach to Culture – Building a Culture That Drives Performance. Washington, DC: Gallup.

[B22] Gkila E. (2017). The Organizational Culture of Cosmote Group, Part of the Growing Telecom Industry and Comparison with that of Banks (Master's Thesis) Patras: Hellenic Open University.

[B23] Hall R. (1993). A framework linking intangible resources and capabiliites to sustainable competitive advantage. Strategic Manage. J. 14, 607–618. 10.1002/smj.4250140804

[B24] Hansen G. S. Wernerfelt B. (1989). Determinants of firm performance: the relative importance of economic and organizational factors. Strategic Manage. J. 10, 399–411. 10.1002/smj.4250100502

[B25] Hellenic Statistical Authority (2022). Statistics: Economy Indices. Available at https://www.statistics.gr/en/statistics/-/publication/SBR01(accessed September 18, 2022).

[B26] Hofstede G. (1980). Culture's Consequences: International Differences in Work- Related Values. London: Sage.

[B27] Hofstede G. Neuijen B. Ohayv D. D. Sanders G (1990). Measuring organizational cultures: a qualitative and quantitative study across twenty cases. Administrat. Sci. Quarterly 35, 286–316. 10.2307/2393392

[B28] Hood C. (1991). A public management for all seasons?. Public Administrat. 69, 3–19. 10.1111/j.1467-9299.1991.tb00779.x

[B29] IAPR (2022). Establisment and Operation of IAPR. Independent Authority of Public Revenue. Available online at: https://www.aade.gr/sites/default/files/2017-04/IAPR.pdf (accessed September 05, 2022).

[B30] Ilie,ş L. Gavrea C. (2008). The link between organizational culture and corporate performance–an overview. Ann. Faculty Econ. 4, 322–329.

[B31] IMF (2022). Greece–Article IV–Consultation. International Monetary Fund, IMFCountry Report No. 22/173.

[B32] Jarnagin C. Slocum J. (2007). Creating corporate cultures through mythopoetic leadership. SMU Cox School Bus. Res. Paper Ser. 7, 4. 10.1016/j.orgdyn.2007.04.004

[B33] Jono S. (2016). Mapping Analysis of the organizational culture by applying the organizational culture assesement instrument (OCAI) at PT. Bank Muamalat Indonesia Bogor Area. Manage. J. Binaniaga 1, 31–40. 10.33062/mjb.v1i01.129

[B34] Jung D. I. Avolio B. J. (1999). Effects of leadership style and followers' cultural orientation on performance in group and individual task conditions. Acad. Manage. J. 42, 208–218. 10.2307/257093

[B35] Kahneman D. Knetsch J. L. Thaler R. H. (1991). Anomalies: the endowment effect, loss aversion, and status quo bias. J. Econ. Perspect. 5, 193–206. 10.1257/jep.5.1.193

[B36] Kalavrezou N. Jin H. (2021). Health Care Reform in Greece: Progress and Reform Priorities. IMF Working Papers. No. 2021/189. 10.5089/9781513588834.001

[B37] Kilmann R. H. Saxton M. J. Serpa R. (1985). Gaining Control of the Corporate Culture. Hoboken: Jossey-Bass, Wiley. p. 1–16.

[B38] Kotter J. P. Heskett J. L. (1992). Corporate Culture and Performance. New York: Free Press.

[B39] Kritikou P. Boufounou P. Toudas K. (2021). Combining economic and cultural perspectives on NPL analysis: a case study. J. Bus. Account. Finance Perspect. 3, 1–16. 10.35995/jbafp3010008

[B40] Lamond D. (2003). The value of Quinn's competing value models, in an Australian context. J. Manage. Psychol. 18, 46–59. 10.1108/02683940310459583

[B41] Lampos N. (2016). The Organizational Culture of Greek Banks (Master's Thesis) Patras: Hellenic Open University.

[B42] Lewis D. (2002). Five years on–the organizational culture saga revisited. Leadership Organiz. Develop. J. 23, 280–287. 10.1108/01437730210435992

[B43] Lovell R. (1995). Managing Change in the New Public Sector. London: Longmann.

[B44] Markatou M. (2012). The role and the importance of the Greek SMEs in the production of innovation. J. Innovat. Bus. Best Pract. 1, 1–10. 10.5171/2012.268692

[B45] Naveed R. T. Jantan A. H. B. Ahmad N. (2016). Organizational culture and organizational change in Pakistani commercial banks. Int. J. Res. 3, 15–18. Available online at: https://www.ijrbsm.org/papers/v3-i8/2.pdf (accessed August 27, 2022).

[B46] Nema P. (2021). Multi-methods approach for information management framework in government control in human resources. J. Contemp. Issues Bus. Government 27, 575–580. 10.47750/cibg.2021.27.03.078

[B47] Ouchi W. G. Wilkins A. L. (1985). Organizational culture. Ann. Rev. Sociol. 11, 457–483. 10.1146/annurev.so.11.080185.002325

[B48] Papadimitriou A. Kargas A. (2012). The relationship between organizational culture and market orientation in the Greek telecommunication companies. Netnomics 13, 1–23. 10.1007/s11066-012-9066-0

[B49] Parker R. Bradley L. (2000). Organisational culture in the public sector: evidence from six organisations. Int. J. Public Sector Manage. 13, 125–141. 10.1108/09513550010338773

[B50] Peters T. J. Waterman R. H. (1982). In Search of Excellence. New York: Harper and Row.

[B51] Pettigrew A. M. (1979). On studying organizational cultures. Administrat. Sci. Quarterly 24, 570–581. 10.2307/2392363

[B52] Quinn R. E. Faerman S. R. Thompson M. P. McGrath M. (1996). Becoming a Master Manager: A Competency Framework. New Jersey: Wiley.

[B53] Rukh H. Qadeer F. (2018). Diagnosing culture of public organization utilizing competing values framework: a mixed methods approach. Pakistan J. Commerce Social Sci. 12, 398–418. Available online at: https://www.econstor.eu/handle/10419/188351 (accessed September 03, 2022).

[B54] Samuelson W. Zeckhauser R. (1988). Status quo bias in decision making. J. Risk Uncertain. 1, 7–59. 10.1007/BF00055564

[B55] Sathe V. (1983). Implications of corporation culture: a manager's guide to action. Organiz. Dyn. 12, 5–23. 10.1016/0090-2616(83)90030-X 10264413

[B56] Schein E. H. (1984). Coming to a new awareness of organizational culture. Sloan Manage. Rev. 25, 3–16.

[B57] Schein E. H. (1990). Organizational culture. Am. Psychol. 45, 109–119. 10.1037/0003-066X.45.2.109

[B58] Schein E. H. (1999). The Corporate Culture Survival Guide, Sense and Nonsense About Culture Change. San Fransisco, California: Jossey Bass.

[B59] Schein E. H. (2010). Organizational Culture and Leadership (Vol. 2). Hoboke: John Wiley and Sons.

[B60] Schraeder M. Tears R. S. Jordan M. H. (2005). Organizational culture in public sector organizations: Promoting change through training and leading by example. Leadership Organiz. Develop. J. 26, 492–502. 10.1108/01437730510617681

[B61] Sinclair A. (1993). Approaches to organisational culture and ethics. J. Bus. Ethics 12, 63–73. 10.1007/BF01845788

[B62] Singh A. (2020). Organizational culture analysis: a study of Indian IT industry using OCAI instrument. Int. J. Manage. 11, 1394–1402. 10.34218/IJM.11.6.2020.128

[B63] Slack N. J. Singh G. (2018). Diagnosis of organizational culture in public sector undertakings undergoing reforms. Public Organiz. Rev. 18, 361–380. 10.1007/s11115-017-0383-5

[B64] Stewart D. (2010). Growing the Corporate Culture. Available online at: https://www.wachovia.com/foundation/v/index.~jsp

[B65] Stewart J. Kimber M. (1996). The transformation of bureaucracy. Aust. J. Public Administrat. 55, 37–48. 10.1111/j.1467-8500.1996.tb01221.x

[B66] Theotokas I. Progoulaki M. (2007). Cultural diversity, manning strategies and management practices in Greek shipping. Maritime Policy Manage. 34, 383–403. 10.1080/03088830701539198

[B67] Titiev M. (1959). Introduction to Cultural Anthropology. New York: Holt.

[B68] Tsakumis G. T. (2007). The influence of culture on accountants' application of financial reporting rules. Abacus 43, 27–48. 10.1111/j.1467-6281.2007.00216.x

[B69] Tsegkou E. (2018). The organizational culture of the Independent Public Revenue Authority. National and Kapodistrian (Master's Thesis) Zografou: University of Athens.

[B70] Tsoukatos E. Rand G. K. (2007). Cultural influences on service quality and customer satisfaction: evidence from Greek insurance. Manag. Service Qual. Int. J. 17, 467–485. 10.1108/09604520710760571

[B71] Umrani W. A. Shah S. M. M. Memon P. A. Samo A. H. (2017). Organizational culture and business performance: an empirical investigation in the Pakistani context. Int. J. Acad. Res. Econ. Manage. Sci. 6, 93–107. 10.6007/IJAREMS/v6-i1/2575

[B72] Van Huy N. Thu N. T. H. Anh N. L. T. Au N. T. H. Cham N. T. Minh P. D.. (2020). The validation of organisational culture assessment instrument in healthcare setting: results from a cross-sectional study in Vietnam. BMC Public Health 20, 1–8. 10.1186/s12889-020-8372-y 32164624 PMC7069212

[B73] Vassilakopoulou V. (2017). The organizational culture of IKA-ETAM (Master's Thesis) Patras: Hellenic Open University.

[B74] Veiga J. F. Yanouzas J. N. (1991). Differences between American and Greek managers in giving up control. Organiz. Stud. 12, 095–108. 10.1177/017084069101200106

[B75] Wallace M. Weese W. J. (1995). Leadership, organizational culture, and job satisfaction in Canadian YMCA organizations. J. Sport Manage. 9, 182–193. 10.1123/jsm.9.2.182

[B76] Zervea E. Apostolakis I. Malliarou M. Sarafis P. (2021). Organizational culture and resistance to change in the Chania general hospital. Upgrade of service quality. Arch. Hellenic Med. 38, 624–634. Available online at: https://www.mednet.gr/archives/2021-5/pdf/624.pdf (accessed August 29, 2022).

[B77] Ziomas D. Theodoroulakis M. (2016). The new Greek pension reform: improving governance and ensuring sustainability. European Commission. ESPN Flash Report 2016/63.

